# Integrating a Top-Gas Recycling and CO_2_ Electrolysis Process for H_2_-Rich Gas Injection and Reduce CO_2_ Emissions from an Ironmaking Blast Furnace

**DOI:** 10.3390/ma15062008

**Published:** 2022-03-08

**Authors:** Yichao Hu, Yinxuan Qiu, Jian Chen, Liangyuan Hao, Thomas Edward Rufford, Victor Rudolph, Geoff Wang

**Affiliations:** 1 School of Chemical Engineering, The University of Queensland, St. Lucia 4072, Australia; h.yichao@uq.edu.au (Y.H.); yinxuan.qiu@uq.edu.au (Y.Q.); t.rufford@uq.edu.au (T.E.R.); v.rudolph@uq.edu.au (V.R.); 2 College of Mechanical and Electrical Engineering, Central South University, Changsha 410083, China; chenj@csu.edu.cn; 3 The Strategy Research Institute, HBIS Group Co., Ltd., Shijiazhuang 050023, China; haoliangyuan@hbisco.com

**Keywords:** blast furnace, hydrogen injection, gas utilisation efficiency, energy consumption, CO_2_ emission

## Abstract

Introducing CO_2_ electrochemical conversion technology to the iron-making blast furnace not only reduces CO_2_ emissions, but also produces H_2_ as a byproduct that can be used as an auxiliary reductant to further decrease carbon consumption and emissions. With adequate H_2_ supply to the blast furnace, the injection of H_2_ is limited because of the disadvantageous thermodynamic characteristics of the H_2_ reduction reaction in the blast furnace. This paper presents thermodynamic analysis of H_2_ behaviour at different stages with the thermal requirement consideration of an iron-making blast furnace. The effect of injecting CO_2_ lean top gas and CO_2_ conversion products H_2_–CO gas through the raceway and/or shaft tuyeres are investigated under different operating conditions. H_2_ utilisation efficiency and corresponding injection volume are studied by considering different reduction stages. The relationship between H_2_ injection and coke rate is established. Injecting 7.9–10.9 m^3^/tHM of H_2_ saved 1 kg/tHM coke rate, depending on injection position. Compared with the traditional blast furnace, injecting 80 m^3^/tHM of H_2_ with a medium oxygen enrichment rate (9%) and integrating CO_2_ capture and conversion reduces CO_2_ emissions from 534 to 278 m^3^/tHM. However, increasing the hydrogen injection amount causes this iron-making process to consume more energy than a traditional blast furnace does.

## 1. Introduction

Traditional blast furnace (BF) iron making relies on carbon and contributes to over 70% of CO_2_ emissions in the iron and steel industry [1]. In a blast furnace, coke is converted into a high-temperature CO gas and performs an exothermic reaction with iron ores, resulting in a large amount of CO and CO_2_ leaving the furnace with top gas. Typically, every tonne of hot metal (tHM) produced from a traditional BF requires about 500 kg/tHM carbon and generates around 1.2 tonnes of CO_2_ emissions [2,3]. Hence, there were various attempts for a clean iron-making process to reduce CO_2_ emissions [4,5,6,7]. One of the approaches is using alternative reductants produced from renewable energies to replace carbon. BF operation with hydrogen as an auxiliary reducing agent was extensively investigated because of its specific advantages over CO [8,9,10,11,12]. Compared to CO reduction that generates CO_2_, reducing iron ores by hydrogen only forms water vapor. Kinetically, hydrogen enables a higher gas flow rate, and a faster reduction in iron ores and productivity than only CO does [13,14]. The higher thermal conductivity of hydrogen helps in heat transfer efficiency between solid and gas phases [15]. In addition, the hydrogen reduction in iron ores suppresses the strong endothermic direct reduction in iron ores. However, hydrogen reduction is thermally more disadvantageous than CO reduction. Due to this endothermic reduction in iron ores by hydrogen, hydrogen addition changes the energy supply of the BF, and it is only useful to a certain extent [16,17,18].

Few previous experiments and mathematical models have investigated the maximal or optimal hydrogen injection to the BF, and results are controversial and need further investigation. A thermogravimetric experiment showed the efficiency of hydrogen on reduction rate is neglected when its content is lower than 5%, and H_2_ content should be 5% to 15% at reduction temperatures between 700 and 1000 °C [13,19]. Wang et al. performed a pulverisation experiment at 900 °C with 70% N_2_ and found that the reduction degree of burdens was more than 90% when H_2_ content was higher than 20% [20]. On the basis of reduction experiments, Lyu et al. reported that the appropriate H_2_ content lies between 5% to 10% in terms of the reduction rate, gas utilisation, and reasonable distribution of the energy in the BF in the CO and H_2_ mixture [17]. Nogami et al. used a multiphase fluid dynamic model to simulate the effect of hydrogen injection with 2.5% oxygen enrichment [8]. They demonstrated that the coke rate decreases linearly, and the maximal hydrogen injection can reach 43.7%.

The application of hydrogen in large-scale BF iron-making processes is limited due to its supply in terms of cost, availability, storage, and transportation [21,22]. The traditional BF contains a low level of hydrogen content because it only generates from the blast air moisture and the volatiles of pulverised coal. One opportunity for BF hydrogen enrichment is injecting hydrogen-rich (so-called H_2_-rich) gas or hydrogen-bearing materials from external sources, including natural gas, fuel oil, coke oven gas (COG), reformed gas, and waste plastics (C_n_H_m_) [23,24,25,26]. Previous studies adopted photocatalysis to produce CO and H_2_ from CO_2_. However, the productivity of photocatalysis is less than 1000 µmol CO/gCO_2_ and 19 µmol H_2_/gCO_2_ [27,28]. It is challenging for photocatalysis to meet the large-scale CO_2_ conversion requirement in the iron-making process. Electrochemical CO_2_ conversion is one option that can recycle carbon into blast furnace gas (BFG) as CO [29]. The high energy demand is considered to be a major difficulty for electrochemical reduction in CO_2_ [30]. However, it provides an added opportunity for a BF because hydrogen is coproduced in the electrolyser, which can be used for iron-ore reduction. In low-temperature electrolysis cells, CO_2_ reduction is carried out in aqueous solutions, and different levels of current density can be used to produce hydrogen at various concentrations [31,32,33]. As an additional benefit, a pure oxygen stream is generated as another byproduct during electrolysis, which can be used directly for oxygen enrichment to the BF [34]. In this study, we use CO_2_ capture and utilisation (CCU) technology to provide a reliable on-site H_2_-rich gas for the BF iron-making process while avoiding CO_2_ emissions. Renewable energy can be coupled to CO_2_ electrolysis to achieve further emission reduction.

This study aims to use a modelling method to predict hydrogen involvement in the BF and determine the CO_2_ emission reduction potential. An alternative method to determine the hydrogen utilisation efficiency is developed. First, thermodynamic and thermal balance models are introduced; then, hydrogen injection is quantitatively studied to determine the optimal injection position and volume by considering key BF performance indices. Lastly, the effect of hydrogen injection on coke consumption, gas utilisation efficiency, and the energy consumption of the iron-making system are investigated.

## 2. Materials and Methods

**Figure 1:**
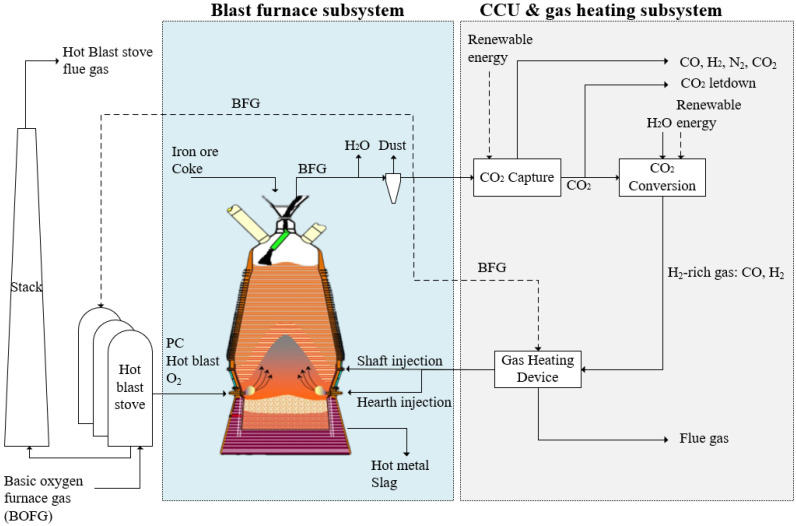
Blast furnace process with CO_2_ capture, conversion, and H_2_-rich gas injection.

Here, we studied the effect of H_2_-rich gas injection through BF tuyeres at the raceway position and/or shaft tuyeres, as shown in Figure 1. The BF is studied as the main subsystem when changing the hydrogen injection condition. The CCU and gas heating subsystem is used as a black box to provide necessary input information to BF. The BFG composition and flow rate from BF are the input for the CCU unit, and the CCU unit provides H_2_-rich reducing gas as the input to the BF. The operating conditions for this BF to produce 1 tonne hot metal (1 tHM) are listed in Table 1 and were kept constant throughout simulations. The overall CO content in the gas injectant was maintained at 200 m^3^/tHM. The productivity of this BF is 238 tHM per hour.

As shown in Figure 1, hot oxygen-enriched blast and pulverised coal is injected through the tuyeres. The upper limit of oxygen enrichment rate for the blast was set at 14% to maintain the stable operation of the large-scale BFs. After drying and dust removal, some BFG is combusted in the hot blast stoves to heat cold blast, and in the gas heating device to provide high-temperature gas injectants. The rest of BFG enters the amine absorption CO_2_ capture unit to provide a CO_2_-rich stream that is processed in an electrochemical CO_2_ conversion unit to produce a CO and H_2_ stream containing, for example, 30% vol. H_2_ and 70% vol. CO. The CO_2_-lean stream from the top of the CO_2_ capture unit contained a mix of CO, H_2_, and N_2_ that is exported to other processes in the integrated steel mill. We did not in this study consider additional separation of CO and H_2_ from the N_2_ in this stream for recycling back to the BF. Oxygen enrichment is required with H_2_-rich gas injection to provide heat to the BF and enrich BFG for CO_2_ capture [35]. The BF can take another advantage from the CCU unit, as the electrolyser produces pure oxygen in another effluent stream. Besides BFG, a small amount of the basic oxygen furnace gas (i.e., BOFG) from steel making is usually combusted as fuel to a hot blast stove. Following assumptions of the BF, CCU unit, gas heating device and hot stove are made in this study:

- Degree of indirect reduction $R_{i}$ depends on the reducing gas concentration of BF bosh gas, which is estimated by an empirical equation, as shown in Equation (1) [36]:

(1) $$R_{i}=0.2777+0.0051\times \%\left(\mathrm{Reducing}\;\mathrm{gas}\right)$$

where %(Reducing gas) is the proportion of H_2_ and CO in the total amount of gas entering the bosh and shaft.

- For the amine absorption CO_2_ capture unit: 30% monoethanolamine (MEA) concentration was used for CO_2_ capture in this study. The capture unit recovered 90% CO_2_ in BFG, and the CO_2_ purity was >99%; the general thermal energy requirement for capture was assumed to be around 1000 kWh/tCO_2_ (3.6 GJ/ tCO_2_) [37,38,39]. Any additional CO_2_ captured and not converted was assumed to be released as per current operation or could be sent to CO_2_ storage routes, shown as CO_2_ letdown in Figure 1.
- The electrochemical CO_2_ conversion unit was treated in the model as a simplified input–output model. Assumptions for the material and energy balance in the CO_2_ conversion unit were based on laboratory demonstration data with additional inputs from literature sources. Briefly, the model was based on multiple two-cell vapour fed electrolyser stacks with the capacity to treat 50 tCO_2_ per day; further details can be found in our other report [33]. The current density of the electrolyser was altered from 2.68 V at 0 A/m^2^ to 3.59 V at 1862 A/m^2^ to produce the H_2_-rich gas with different H_2_/CO compositions.
- The electricity consumption for CO_2_ conversion is proportional to the H_2_ generation, which can be estimated as in Equation (2) [33]:

(2) $$E_{\mathrm{conv}}=10.75V_{H_{2}\mathrm{gen}}+1282$$

where $E_{\mathrm{conv}}$ is the power required for the CO_2_ conversion unit, kWh/tHM; $V_{H_{2}\mathrm{gen}}$ is the amount of H_2_ generated by the conversion unit, m^3^/tHM;

- efficiency of the gas heating device was 85%;
- The hot blast stove system uses two stoves on-gas and one stove on-blast, and the efficiency of the hot blast stoves was 75%.

As indicated in Figure 1, CO_2_ capture and conversion units use renewable energy to avoid their own CO_2_ emissions. The type of renewable power used by the industry depends on availability and cost, such as solar power [40,41]. Besides solar power, industries can use thermal–electrical materials to recover a large amount of waste heat in an integrated steel mill to provide electricity for CCU units [42]. In addition, using the lower heating value energy to generate electricity in the steel mill and the on-site power plant can help to minimise the renewable power periodic availability problem.

To achieve the objectives of this study, two mathematical models were developed. As a reducing agent, H_2_-rich gas needs to fulfil the thermodynamic requirement of the reduction reaction to capture oxygen in iron ores. H_2_-rich gas also needs to provide enough heat in the shaft for keeping an effective reduction process. First, hydrogen behaviour in different parts of the BF was analysed. A thermodynamic model for hydrogen reduction was built to determine hydrogen utilisation efficiency. This model provides a guideline of the proper hydrogen injection concentration. Then, a thermal balance model was used to limit the hydrogen injection temperature and volume.

The optimal hydrogen injection amount and position were determined by increasing the reduction potential in the coke consumption and CO_2_ emissions, increasing gas utilisation efficiency and lowering the energy consumption. A static mass balance model of the BF was used to calculate the above parameters.

### 2.1. Thermodynamic Calculations of H_2_-Rich Gas Injection BF

The reduction behaviours of injected gas are discussed in different parts of the BF to determine the reducing gas utilisation.

#### 2.1.1. Raceway

In the BF raceway, the main reactions considered in this study were carbon combustion, coke solution loss reaction between coke and CO_2_, water–gas reaction between coke and moisture in the hot blast, which can be described as shown in Equations (3)–(5):

(3) $$\mathrm{Solution}\;\mathrm{loss}\;\mathrm{reaction}\;C\;\left(s\right)+{\mathrm{CO}}_{2}\;\left(g\right)\to 2\mathrm{CO}\;\left(g\right)\;\Delta H^{0}=172,430\;\mathrm{kJ}/\mathrm{kmol}$$

(4) $$\mathrm{Water}–\mathrm{gas}\;\mathrm{reaction}\;C\;\left(s\right)+H_{2}O\left(g\right)\to \mathrm{CO}\;\left(g\right)+H_{2}\;\left(g\right)\Delta H^{0}=124,190\;\mathrm{kJ}/\mathrm{kmol}\;$$

(5) $$\;\Delta G^{0}=134,542-142.28T$$

With excess coke existing in the BF bottom, it could be assumed that CO and H_2_ combustion was negligible. This could be justified by the assumption that, if a small amount of H_2_ reacts with O_2_ to form water in the raceway, the generated water vapour reacts with coke and turn back to H_2_. Therefore, this process can be simplified as heating the hearth gas injectant, as shown in Equation (6):

(6) $$\Delta Q_{\mathrm{hearth}}=\int_{25}^{T_{\mathrm{hearth}}}V_{\mathrm{hearth}}\cdot C_{p\_\mathrm{hearth}}\mathrm{dt}$$

where $T_{\mathrm{hearth}}$ is hearth gas injection temperature, °C; $C_{p\_\mathrm{hearth}}$ denotes specific heat capacity of the gas injected to BF, kJ/m^3^·°C; $V_{\mathrm{hearth}}$ is hearth injection volume, m^3^/tHM; and $\Delta Q_{\mathrm{hearth}}$ is the sensible heat carried by the gas injected to BF, kJ/tHM.

#### 2.1.2. Dripping, Cohesive, and High-Temperature Zones over 1000 °C

The primary reaction in the dripping zone is a direct reaction between coke and FeO. Hot gas containing H_2_ and CO that passes through the cohesive zone reacts with molten FeO or semi-molten FeO to form H_2_O and CO_2_. As the temperature was over 1350 °C in the dripping zone, the amount of CO_2_ was negligible due to the solution loss reaction. At the high-temperature zone over 1000 °C, some reduced FeO and Fe_3_O_4_ were still in the solid state, and H_2_ could pass through their surface. Almost all the H_2_O produced by H_2_ reduction rapidly participates in water–gas reaction at the presence of coke to form CO and H_2_ over 1273 K (1000 °C). Therefore, the reduction reaction in this section was essentially the direct reduction in iron by coke. H_2_ injected through the tuyeres at the raceway mainly catalyses direct reduction and heats the molten or semi-molten burden.

#### 2.1.3. Shaft Zone Temperature between 800 and 1000 °C

According to the thermodynamics of iron oxide reduction and dynamics studies, H_2_ has better reducing capability than that of CO above 800 °C [43,44]. At the same time, the extent of coke solution loss reaction and the water–gas reaction was less than that in the higher temperature zone. In this temperature zone, H_2_ reacts with various iron oxides to generate H_2_O, and the formed H_2_O is not gasified into H_2_ by carbon completely. Therefore, it is the primary zone to improve H_2_ utilisation efficiency.

The H_2_-rich reducing gas utilisation rate and volume requirement vary in different ferric oxides reduction stages. The iron oxides reduction reactions are at a nonequilibrium state in the BF. When the temperature is above 570 °C, the reduction in ferric oxides by CO and H_2_ in the BF occurs in the following sequences: 1/2 Fe_2_O_3_ → 1/3 Fe_3_O_4_ (Stage I) → FeO (Stage II) → Fe (Stage III) [45]. The gas produced by the reduction in the latter stage is the reducing gas for the previous stage. Heat is gradually transferred to solid materials during the gas ascending. At the same time, part of reducing gas reacts with iron oxides and converts into CO_2_ and H_2_O, and finally forms top gas at around 150 to 250 °C when leaving the BF. There are 25% of the total oxygen elements removed during the reduction of Fe_3_O_4_ into FeO, and the remaining 75% of oxygen elements were removed in reducing FeO to Fe. Therefore, the reduction process from FeO to Fe is the key step. The required reducing gas amount is n kmol for CO, and m kmol for H_2_ to produce 1 kmol iron. The value of n and m is the excess coefficient. The reduction reactions and thermodynamic parameters in Stage III for CO and H_2_ are expressed as in Equations (7)–(12), and Equations (13)–(17), respectively [46]:

(7) $$\mathrm{Stage}\;\mathrm{III}:\;\;\mathrm{FeO}\;\left(s\right)+\mathrm{nCO}\;\left(g\right)\to \mathrm{Fe}\;\left(s\right)+{\mathrm{CO}}_{2\;}\left(g\right)+\left(n-1\right)\mathrm{CO}\;\left(g\right)\;$$

$$\Delta H_{3\mathrm{CO}}^{0}=-13,190\;\mathrm{kJ}/\mathrm{kmol}$$

(8) $$\Delta G_{3\mathrm{CO}}^{0}=-22,800-24.26T,\;\mathrm{kJ}/\mathrm{kmol}$$

(9) $${\mathrm{lnK}}_{\mathrm{IIICO}}=-\frac{\Delta G_{3\mathrm{CO}}^{0}}{\mathrm{RT}}=-\frac{-22,800-24.26T}{\mathrm{RT}}\;$$

(10) $$K_{\mathrm{IIICO}}=\frac{p_{{\mathrm{CO}}_{2}}}{p_{\mathrm{CO}}}=\frac{\varphi_{{\mathrm{CO}}_{2}}\times P^{0}}{\varphi_{\mathrm{CO}}\times P^{0}}=\frac{1}{n-1}$$

where K is the reaction equilibrium constant; φ denotes the fraction of gas component.

The minimal CO required for Stage III is described in Equation (11):

(11) $$n=1+\frac{1}{K_{\mathrm{IIICO}}}$$

The utilisation efficiency of CO in Stage III, $\eta_{3}{}_{\mathrm{CO}}$, is described in Equation (12):

(12) $$\eta_{3}{}_{\mathrm{CO}}=\frac{\varphi_{3}{}_{{\mathrm{CO}}_{2}}}{\varphi_{3}{}_{\mathrm{CO}}+\varphi_{3}{}_{{\mathrm{CO}}_{2}}}=\frac{1}{\frac{\varphi_{3}{}_{\mathrm{CO}}}{\varphi_{3{\mathrm{CO}}_{2}}}+1}=\frac{1}{\frac{1}{K_{3}{}_{\mathrm{CO}}}+1}=\frac{K_{\mathrm{III}}{}_{\mathrm{CO}}}{1+K_{\mathrm{III}}{}_{\mathrm{CO}}}=\frac{1}{n}$$

(13) $$\mathrm{FeO}\;\left(s\right)+{\mathrm{mH}}_{2}\;\left(g\right)\to \mathrm{Fe}\;\left(s\right)+H_{2}O\left(g\right)+\left(m-1\right)H_{2}\;\left(g\right)$$

$$\Delta H_{3H_{2}}^{0}=28,010\;\mathrm{kJ}/\mathrm{kmol}$$

(14) $$\Delta G_{3H_{2}}^{0}=23,430-16.16T,\;\mathrm{kJ}/\mathrm{kmol}$$

(15) $${\mathrm{lnK}}_{{\mathrm{IIIH}}_{2}}=-\frac{\Delta G_{3H_{2}}^{0}}{\mathrm{RT}}=-\frac{23,430-16.16T}{\mathrm{RT}}$$

The minimal H_2_ required for Stage III is calculated as in Equation (16):

(16) $$m=1+\frac{1}{K_{{\mathrm{IIIH}}_{2}}}$$

The utilisation efficiency of H_2_ in Stage III, $\eta_{3}{}_{H_{2}}$ is described in Equation (17):

(17) $$\eta_{3}{}_{H_{2}}=\frac{\varphi_{3}{}_{H_{2}O}}{\varphi_{3}{}_{H_{2}}+\varphi_{3}{}_{H_{2}O}}=\frac{K_{\mathrm{III}}{}_{H_{2}}}{1+K_{\mathrm{III}}{}_{H_{2}}}=\frac{1}{m}$$

According to the theoretical thermochemical calculations, 50% of H_2_ and CO participates in the water–gas shift reaction Equation (18) between 600 and 1400 °C [47]. Therefore, the heat consumed by the water–gas shift reaction at temperatures above 820 °C is balanced by the heat generated at the temperature below 820 °C, as calculated by Equation (19). However, H_2_ promotes iron ore reduction by CO via the water gas shift reaction when the temperature is over 820 °C [48]. The CO_2_ generated reacts with H_2_ to reform CO, which participates in FeO reduction reaction again and improve the utilisation efficiency of CO.

(18) $$\mathrm{Water}–{\mathrm{gas}\;\mathrm{shift}\;\mathrm{reaction}\;H}_{2}O\left(g\right)+\;\mathrm{CO}\;\left(g\right)=H_{2}\;\left(g\right)+{\mathrm{CO}}_{2}\;\left(g\right)$$

$$\Delta H^{0}=-41,325\;\mathrm{kJ}/\mathrm{kmol}$$

(19) $$\Delta G^{0}=-33,447+30.56T,\;\mathrm{kJ}/\mathrm{kmol}$$

The heat effect of FeO reduction by H_2_ and CO gas is calculated by Equation (20):

(20) $$\Delta H=\frac{X_{\mathrm{CO}}\eta_{3\mathrm{CO}}\Delta H_{3\mathrm{CO}}^{0}+X_{H2}\eta_{3H_{2}}\Delta H_{3H_{2}}^{0}}{X_{\mathrm{CO}}\eta_{3\mathrm{CO}}+X_{H2}\eta_{3H_{2}}}$$

where $X_{i}$ is the proportion of CO or H_2_ in the reducing gas entering the BF shaft.

The gas produced by the reduction in Stage III is the reducing gas for Stage II. The reduction reactions and thermodynamic parameters in Stage II for CO and H_2_ are expressed in Equations (21)–(28):

(21) $$\mathrm{Stage}\;\mathrm{II}:\;\frac{1}{3}{\mathrm{Fe}}_{3}O_{4}\;\left(s\right)+\left(n-1\right)\mathrm{CO}\;\left(g\right)+{\mathrm{CO}}_{2\;}\left(g\right)\to \mathrm{FeO}\;\left(s\right)+\frac{4}{3}{\mathrm{CO}}_{2}\;\left(g\right)+\left(n-\frac{4}{3}\right)\mathrm{CO}\;\left(g\right)\;\Delta H_{2\mathrm{CO}}^{0}=22,400\;\mathrm{kJ}/\mathrm{kmol}\;$$

(22) $$\Delta G_{2\mathrm{CO}}^{0}=35,380-40.16T,\;\mathrm{kJ}/\mathrm{kmol}$$

(23) $$\frac{1}{3}{\mathrm{Fe}}_{3}O_{4}\;\left(s\right)+\left(m-1\right)H_{2}\left(g\right)+H_{2}O\;\left(g\right)\to \mathrm{FeO}\;\left(s\right)+\frac{4}{3}H_{2}O\;\left(g\right)+\left(m-\frac{4}{3}\right)H_{2}\left(g\right)\;\Delta H_{2H_{2}}^{0}=63,600\;\mathrm{kJ}/\mathrm{kmol}$$

(24) $$\Delta G_{2H_{2}}^{0}=71,940-73.62T,\;\mathrm{kJ}/\mathrm{kmol}$$

(25) $${\mathrm{lnK}}_{\mathrm{IICO}}=-\frac{\Delta G_{2\mathrm{CO}}^{0}}{\mathrm{RT}}=-\frac{35,380-40.16T}{\mathrm{RT}}\;$$

(26) $${\mathrm{lnK}}_{{\mathrm{IIH}}_{2}}=-\frac{\Delta G_{2H_{2}}^{0}}{\mathrm{RT}}=-\frac{71,940-73.62T}{\mathrm{RT}}$$

(27) $$K_{\mathrm{IICO}}=\frac{\varphi_{{\mathrm{CO}}_{2}}\times P^{0}}{\varphi_{\mathrm{CO}}\times P^{0}}=\frac{\frac{4}{3}}{n-\frac{4}{3}}$$

(28) $$K_{{\mathrm{IIH}}_{2}}=\frac{\frac{4}{3}}{m-\frac{4}{3}}$$

The minimal CO and H_2_ volume required for Stage II is calculated as shown in Equations (29) and (30), respectively:

(29) $$n=\frac{4}{3}\left(1+\frac{1}{K_{\mathrm{IICO}}}\right)$$

(30) $$m=\frac{4}{3}\left(1+\frac{1}{K_{{\mathrm{IIH}}_{2}}}\right)$$

The utilisation efficiency of CO and H_2_ in Stage II is calculated as shown in Equations (31) and (32), respectively:

(31) $$\eta_{2}{}_{\mathrm{CO}}=\frac{\varphi_{2}{}_{{\mathrm{CO}}_{2}}}{\varphi_{2}{}_{\mathrm{CO}}+\varphi_{2}{}_{{\mathrm{CO}}_{2}}}=\frac{K_{\mathrm{II}}{}_{\mathrm{CO}}}{1+K_{2}{}_{\mathrm{CO}}}=\frac{4}{3n}$$

(32) $$\eta_{2}{}_{H_{2}}=\frac{\varphi_{2}{}_{H_{2}O}}{\varphi_{2}{}_{H_{2}}+\varphi_{2}{}_{H_{2}O}}=\frac{K_{\mathrm{II}}{}_{H_{2}}}{1+K_{2}{}_{H_{2}}}=\frac{4}{3m}$$

In the first stage of iron ores reduction, the transformation of Fe_2_O_3_ to Fe_3_O_4_ is very rapid due to the very high equilibrium constant of Fe_2_O_3_ reduction above 600 K, as shown in Equations (33) and (35):

(33) $$\mathrm{Stage}\;I:\;\;\frac{1}{2}{\mathrm{Fe}}_{2}O_{3}\;\left(s\right)+\left(n-\frac{4}{3}\right)\mathrm{CO}\;\left(g\right)+\frac{4}{3}{\mathrm{CO}}_{2}\;\left(g\right)\;\to \frac{1}{3}{\mathrm{Fe}}_{3}O_{4}\;\left(s\right)+\frac{3}{2}{\mathrm{CO}}_{2}\left(g\right)+\left(n-\frac{3}{2}\right)\mathrm{CO}\left(g\right)\;\Delta H_{1\mathrm{CO}}^{0}=-43,280\;\mathrm{kJ}/\mathrm{kmol}$$

(34) $$\Delta G_{1\mathrm{CO}}^{0}=-52,131-41.0T,\;\mathrm{kJ}/\mathrm{kmol}$$

(35) $$\frac{1}{2}{\mathrm{Fe}}_{2}O_{3}\;\left(s\right)+\left(m-\frac{4}{3}\right)H_{2}\;\left(g\right)+\frac{4}{3}H_{2}O\;\left(g\right)\to \frac{1}{3}{\mathrm{Fe}}_{3}O_{4}\;\left(s\right)+\frac{3}{2}H_{2}O\left(g\right)+\left(m-\frac{3}{2}\right)H_{2}\;\left(g\right)\;\Delta H_{1H_{2}}^{0}=-21,810\;\mathrm{kJ}/\mathrm{kmol}$$

(36) $$\Delta G_{1H_{2}}^{0}=-15,547-74.4T,\;\mathrm{kJ}/\mathrm{kmol}$$

The gas produced by the reduction in Stage II provides the reducing gas for Stage I. These reactions only require a low concentration of reducing gas to proceed. The minimal CO and H_2_ volume required for Stage I is shown in Equations (37) and (38), respectively. With utilisation efficiency close to 100%, Fe_2_O_3_ reduction is an irreversible reaction.

(37) $$n=\frac{3}{2}\left(1+\frac{1}{K_{\mathrm{ICO}}}\right)$$

(38) $$m=\frac{3}{2}\left(1+\frac{1}{K_{{\mathrm{IH}}_{2}}}\right)$$

The utilisation efficiency of CO and H_2_ in Stage I is described by Equations (39) and (40), respectively:

(39) $$\eta_{1}{}_{\mathrm{CO}}=\frac{\varphi_{2}{}_{{\mathrm{CO}}_{2}}}{\varphi_{2}{}_{\mathrm{CO}}+\varphi_{2}{}_{{\mathrm{CO}}_{2}}}=\frac{3}{2n}$$

(40) $$\eta_{1}{}_{H_{2}}=\frac{\varphi_{2}{}_{H_{2}O}}{\varphi_{2}{}_{H_{2}}+\varphi_{2}{}_{H_{2}O}}=\frac{3}{2m}$$

The overall gas utilisation efficiency for H_2_-rich reducing gas in the BF is calculated as in Equation (41) below:

(41) $$\eta =\frac{\varphi_{{\mathrm{CO}}_{2}}+\varphi_{H_{2}O}}{\varphi_{\mathrm{CO}}+\varphi_{{\mathrm{CO}}_{2}}+\varphi_{H_{2}}+\varphi_{H_{2}O}}=X_{\mathrm{CO}}\eta_{\mathrm{CO}}+X_{H_{2}}\eta_{H_{2}}$$

Assuming the water generated in the Fe_2_O_3_ reduction is reacted with CO, in which H_2_ performs only as a catalyst of CO reduction of Fe_2_O_3_. The water in top gas is determined by H_2_ utilisation efficiency in FeO and Fe_3_O_4_, which was calculated as in Equation (42):

(42) $$V_{H_{2}O}=\sum V_{H_{2}}\eta_{3H_{2}}+\sum V_{H_{2}}\left(1-\eta_{3H_{2}}\right)\eta_{2H_{2}}+\sum V_{H_{2}}\left(1-\eta_{3H_{2}}\right)\left(1-\eta_{2H_{2}}\right)\eta_{1H_{2}}$$

Since FeO reduction is the key step, the theoretical overall H_2_ utilisation efficiency was calculated as shown in Equation (43). The highest theoretical H_2_ utilisation efficiency can be obtained with the minimal H_2_ requirement value on the basis of the thermodynamic requirement in Stage III, and this highest value is determined by temperature. Due to thermal restrictions and excess H_2_ injected, the actual gas utilisation efficiency can only approach this theoretical value. The actual thermodynamic utilisation efficiency of H_2_ is a function of the amount of H_2_ introduced to the BF, as shown in Equation (43):

(43) $$\eta_{H_{2}}=\eta_{3H_{2}}+\left(1-\eta_{3H_{2}}\right)\eta_{2H_{2}}+\left(1-\eta_{3H_{2}})(1-\eta_{2H_{2}}\right)\eta_{1H_{2}}\;=\frac{1}{m}+\left(1-\frac{1}{m}\right)\frac{4}{3m}+\left(1-\frac{1}{m}\right)\left(1-\frac{4}{3m}\right)\frac{3}{2m}$$

As the FeO reduction is the key step in the indirect reduction process, the thermodynamic requirement of gas entering the BF shaft to produce 1 tHM is calculated as in Equations (44) and (45):

(44) $$V_{\mathrm{bosh}\_\mathrm{shaft}}=\frac{1000{\left(\mathrm{Fe}\right)}_{\mathrm{HM}}\left(1-R_{d}\right)}{\eta_{3}\times \%\left(\mathrm{Reducing}\;\mathrm{gas}\right)}\times \frac{22.4}{56}$$

(45) $$R_{d}=1-R_{i}$$

where $V_{\mathrm{bosh}\_\mathrm{shaft}}$ is the amount of gas raised from BF bosh after direct reduction and the gas injected through the shaft tuyeres, m^3^/tHM; ${\left[\mathrm{Fe}\right]}_{\mathrm{HM}}$ is the proportion of iron content in hot metal; $R_{d}$ is the degree of direct reduction.

### 2.2. Thermal Calculations of H_2_-Rich Gas Injection BF

As the heat carrier, the H_2_-rich gas injected through raceway tuyeres needs to compensate for the required energy in the lower furnace and maintain the theoretical combustion temperature at a reasonable range. The gas injected through the shaft also needs to satisfy the heat requirement in the upper furnace. The energy of H_2_-rich gas includes the oxidation heat release from the iron ore reduction and sensible heat. The oxidation heat release depends on gas utilisation efficiency and gas composition. The injection temperature determines the sensible heat. Thermal calculations for determining the amount of H_2_-rich gas were developed by a static mass and energy model of the iron-making process.

The thermal balance for this iron-making process is developed in the lower and upper furnaces, divided by the shaft gas injection position. In this work, the lower furnace included BF raceway, dripping zone, and cohesive zone. The thermal balance of the lower furnace is shown in Equation (46) below:

(46) $$Q_{\mathrm{cc}}+Q_{\mathrm{blast}}+Q_{\mathrm{hearth}}+Q_{\mathrm{coke}}+Q_{\mathrm{ore}}\;=H_{{\mathrm{CO}}_{2}}+H_{H_{2}\mathrm{Ode}}+H_{\mathrm{PCde}}+Q_{\mathrm{bosh}}+H_{\mathrm{dA}}+H_{\mathrm{dFe}}+H_{S}+Q_{\mathrm{HM}}+Q_{\mathrm{slag}}+Q_{\mathrm{loss}\_l}$$

where the heat income in the lower furnace includes: $Q_{\mathrm{cc}}$ = combustion heat of coke and pulverised coal in front of tuyeres, kJ/tHM; $Q_{\mathrm{blast}}$ = sensible heat of the hot blast, kJ/tHM; $Q_{\mathrm{hearth}}$ = sensible heat of H_2_-rich gas injection to the hearth, kJ/tHM; $Q_{\mathrm{coke}}$ = heat of the coke brings to the lower part of BF, kJ/tHM; $Q_{\mathrm{ore}}$ = sensible heat of the iron ores into the lower part of the BF, kJ/tHM; and the heat expenditure includes: $H_{\mathrm{CO}2}$ = heat consumption of solution loss reaction due to the possible CO_2_ in the hearth injection gas, kJ/tHM; $H_{H_{2}\mathrm{Od}}$ = heat consumption of water decomposition in front of tuyeres, kJ/tHM; $H_{\mathrm{PCde}}$ = heat consumption of pulverised coal decomposition in front of tuyeres, kJ/tHM; $Q_{\mathrm{bosh}}\;$= heat brought to the shaft by bosh gas, kJ/tHM; $H_{\mathrm{dA}}$ = heat consumption by direct reduction of alloy element, kJ/tHM; $H_{\mathrm{dFe}}$ = heat consumption by direct reduction of FeO, kJ/tHM; $H_{S}$ = heat consumption by desulphurisation, kJ/tHM; $Q_{\mathrm{HM}}$ = sensible heat of hot metal, kJ/tHM; $Q_{\mathrm{slag}}$ = sensible heat of slag, kJ/tHM; and $Q_{\mathrm{loss}\_l}$ = heat loss in the lower furnace, kJ/tHM.

With H_2_-rich gas injection to BF hearth, the raceway adiabatic flame temperature (RAFT) is calculated as in Equation (47):

(47) $$\mathrm{RAFT}\;=\frac{Q_{\mathrm{coke}}+Q_{\mathrm{blast}}+Q_{\mathrm{hearth}}+Q_{\mathrm{cc}}-Q_{H2\mathrm{Ode}}-Q_{\mathrm{PCde}}-H_{{\mathrm{CO}}_{2}}\;}{V_{H_{2}}^{\mathrm{raceway}}\times C_{H_{2}}+V_{\mathrm{CO}}^{\mathrm{raceway}}\times C_{\mathrm{CO}}+V_{N_{2}}^{\mathrm{raceway}}\times C_{N_{2}}}$$

where $Q_{\mathrm{coke}}$ is the heat brought to the raceway by coke, kJ/tHM; $V_{i}^{\mathrm{raceway}}$ is the gas volumes of H_2_, CO and N_2_ in the raceway, m^3^/tHM, respectively.

The energy input and output of the BF shaft can be expressed as in Equations (48) and (49), respectively:

(48) $$Q_{\mathrm{shaft}}+Q_{\mathrm{bosh}}+Q_{\mathrm{ind}}+Q_{\mathrm{ore}\_\mathrm{shaft}}=Q_{\mathrm{top}}+Q_{\mathrm{coke}}+Q_{\mathrm{ore}}+Q_{\mathrm{loss}\_s}$$

(49) $$Q_{\mathrm{ind}}=Q_{{\mathrm{Fe}}_{2}O_{3}}+Q_{{\mathrm{Fe}}_{3}O_{4}}+Q_{\mathrm{FeO}}$$

where $Q_{\mathrm{shaft}}$ is the heat carried by H_2_-rich gas injected into the shaft, kJ/tHM; $Q_{\mathrm{ind}}$ is the heat generation by iron oxides reduction by H_2_ and CO, kJ/tHM; $Q_{\mathrm{ore}\_\mathrm{shaft}}$ is the sensible heat carried by iron ores entering the BF top, kJ/tHM; $Q_{\mathrm{top}}$, kJ/tHM is heat loss in the shaft, kJ/tHM.

The heats provided by the reduction reactions from Fe_2_O_3_ to Fe_3_O_4_, Fe_3_O_4_ to FeO, and FeO to Fe by CO and H_2_ are calculated as from Equations (50)–(52), respectively:

(50) $$Q_{{\mathrm{Fe}}_{2}O_{3}}=56\times \frac{m_{\mathrm{ore}}\times \frac{w{\left({\mathrm{Fe}}_{2}O_{3}\right)}_{\mathrm{ore}}}{160}}{3}\left(r_{\mathrm{ico}}\times \Delta H_{1\mathrm{CO}}^{0}+r_{{\mathrm{iH}}_{2}}\times \Delta H_{1H_{2}\;}^{0}\right)$$

(51) $$Q_{{\mathrm{Fe}}_{3}O_{4}}=56\times \frac{2\times m_{\mathrm{ore}}\times \frac{w{\left({\mathrm{Fe}}_{2}O_{3}\right)}_{\mathrm{ore}}}{160}}{3}\left(r_{\mathrm{ico}}\times \Delta H_{2\mathrm{CO}}^{0}+r_{{\mathrm{iH}}_{2}}\times \Delta H_{2H_{2}\;}^{0}\right)$$

(52) $$\begin{array}{c} Q_{\mathrm{FeO}}=56\times \left(1-R_{d}\right)\left(\frac{2\times m_{\mathrm{ore}}\times w{\left({\mathrm{Fe}}_{2}O_{3}\right)}_{\mathrm{ore}}}{160}+\frac{m_{\mathrm{ore}}\times w{\left(\mathrm{FeO}\right)}_{\mathrm{ore}}}{72}\right)\\ \left(r_{\mathrm{ico}}\times \Delta H_{3\mathrm{CO}}^{0}+r_{{\mathrm{iH}}_{2}}\times \Delta H_{3H_{2}\;}^{0}\right) \end{array}$$

where $r_{\mathrm{ico}}$ and $r_{{\mathrm{iH}}_{2}}$ are the degree of indirect reduction by CO and H_2_, as shown in Equations (53) and (54), respectively:

(53) $$r_{{\mathrm{iH}}_{2}}=\frac{V_{H2O}}{22.4\times 1000{\left(\mathrm{Fe}\right)}_{\mathrm{HM}}}\times 56$$

(54) $$r_{\mathrm{iCO}}={\;R}_{i}-r_{{\mathrm{iH}}_{2}}$$

## 3. Results and Discussion

### 3.1. Results of the Thermodynamic Model

**Figure 2:**
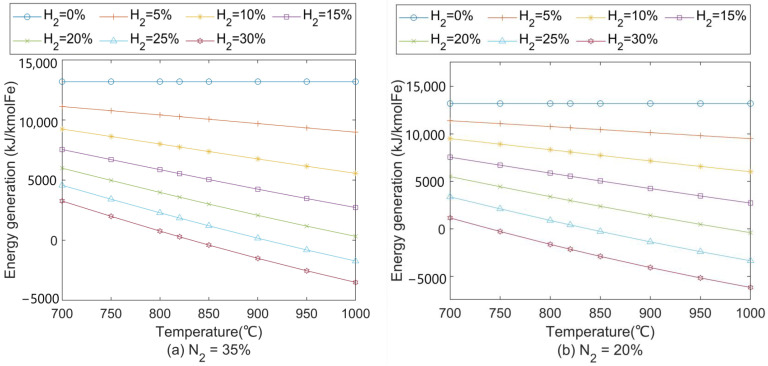
Heat effect of FeO reduction in H_2_–CO–N_2_ gas mixture at (**a**) 35% N_2_ and (**b**) 20% N_2_. H_2_ content entering blast furnace shaft is indicated by different colours.

At a medium oxygen enrichment rate (9%), the nitrogen content in the BF shaft is calculated at around 35%. Figure 2a shows the heat effect results based on Equation (20). The FeO reduction reaction transforms from an exothermic into an endothermic process when H_2_ content increases to 25% around 900 °C. A similar phenomenon shows up when the H_2_ reaches 20% around 1000 °C. With more H_2_ participating in the reduction at high temperatures, it causes a severe negative effect on the thermal energy supply to the BF. Therefore, the shaft gas injection temperature should not be too high to reduce its endothermic heat effect and require less preheating in the gas preheating device. The higher oxygen enrichment (less N_2_ content) enables higher H_2_ content in the BF, as shown in Figure 2b. It is suggested that with 20% N_2_ entering the BF shaft, the H_2_ content should be lower than 25% to avoid too much heat consumption by its reduction reaction.

**Figure 3:**
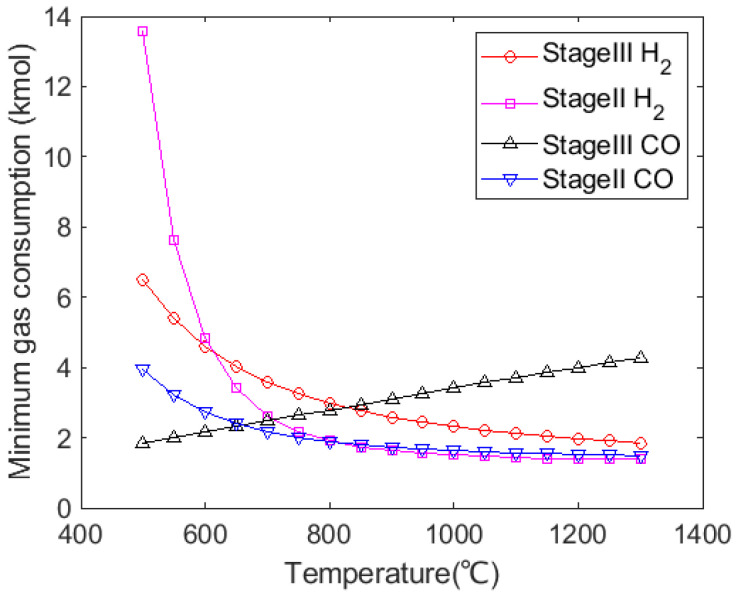
Variation in minimal gas consumption for iron ores reduction in pure H_2_ or CO with temperature.

According to Equations (11), (16), (29) and (30), the theoretical minimal H_2_ and CO requirement with temperature are shown in Figure 3. From this figure, Stage III for iron ores reduction requires more H_2_ when the temperature is over 625 °C. Stage III requires more CO over 650 °C. In this study, since the gas injection temperature is kept above 820 °C to ensure the high reducing capability of H_2_, the thermodynamic key step for iron ores reduction is Stage III, and the other stages are proceeded with excess reducing gas. Compared with Fe_3_O_4_, FeO reduction requires more reducing gas to proceed, which determines the minimal amount of gaseous mixture. Fe_3_O_4_ and Fe_2_O_3_ reductions are carried out with excess reducing gas. In addition, the amount of H_2_ required for FeO reduction at high temperatures is less than the amount of CO required in the reaction. Thermodynamically, injecting H_2_ content to replace some amount of CO would reduce the total amount of gas mixture and reduce the fuel requirement for the gas preheating. However, CO content in the BF should be enough to meet its thermal condition.

**Figure 4:**
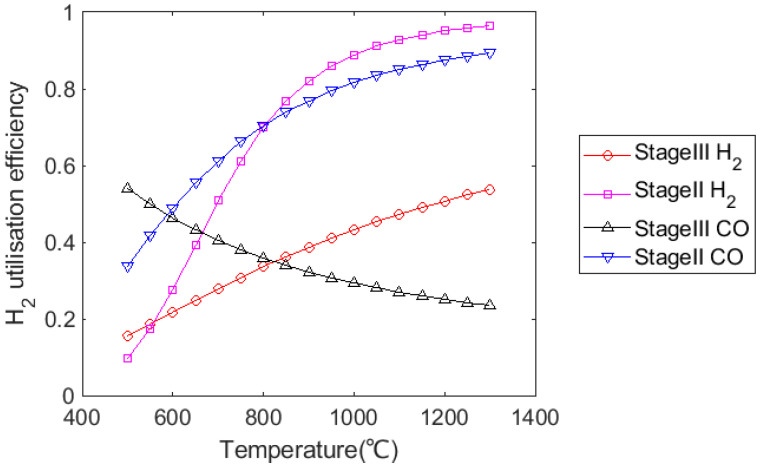
Variation in gas utilisation efficiency for iron ores reduction in pure H_2_ or CO with temperature.

According to Figure 4, gas utilisation efficiency for H_2_ and CO was similar in Stage II when the temperature was above 820 °C, but H_2_ utilisation efficiency was much higher than that for CO in Stage III. Although the utilisation efficiency of CO decreases as temperature increases, the FeO reduction reaction would still be promoted with increasing H_2_ content due to the effect of the water–gas shift reaction.

**Figure 5:**
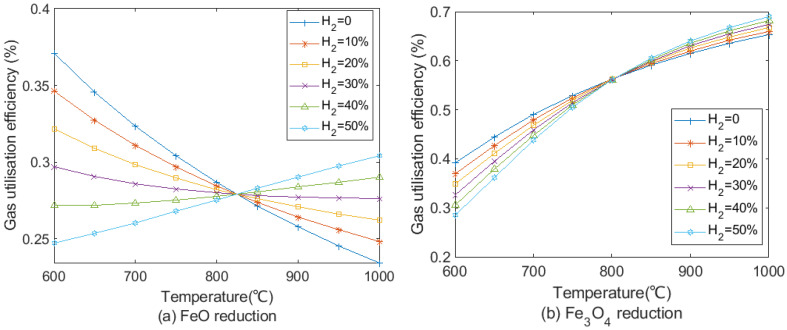
Gas utilisation efficiency at different H_2_/CO ratio with temperature in (**a**) FeO and (**b**) Fe_3_O_4_ reductions.

On the basis of the calculation from Equation (41), gas utilisation efficiency at different H_2_ contents in the reducing gas for FeO and Fe_3_O_4_ reduction is shown in Figure 5.

Figure 5a presents the gas utilisation efficiency of FeO reduction. Due to the endothermic reaction of the H_2_ reduction, utilisation efficiency of H_2_-rich reducing gas at 1000 °C for the FeO reduction increased from 23% with no H_2_ to around 30% with 50% H_2_ by the equal interval. When H_2_ content was less than 30% in reducing gas, gas utilisation efficiency in FeO reduction decreased with the increase in temperature. In contrast, gas utilisation efficiency rose with temperature when H_2_ content is more than 40% in reducing gas. Hence, H_2_ content in the reducing gas should not be too low to hinder improvement in the gas utilisation efficiency. As shown in Figure 5b, since Fe_3_O_4_ reductions by CO and H_2_ are endothermic, gas utilisation efficiency increases with temperature. The effect of increases in H_2_ content for Fe_3_O_4_ reduction is less than FeO reduction in terms of gas utilisation efficiency. At 1000 °C, gas utilisation efficiency increases by less than 4% when H_2_ content increases from 0% to 50%. The shaft gas injection temperature should be higher than 820 °C to promote gas utilisation in FeO and Fe_3_O_4_ reduction, especially focusing on FeO reduction.

### 3.2. BF Simulation Conditions and Validation

Without gas injection, the model developed in this work can be used for a traditional BF. The measured data collected from a 2500 m^3^ BF were used to validate this proposed model. The comparison of the industrial data and the model predictions is summarised in Table 2. The coke rate and top gas components were compared because they are essential measurable parameters that indicate the overall performance of an iron-making BF. The chemical composition of raw material data is shown in Table 3, Table 4 and Table 5. In general, results in this simulation show a similar trend as in practice, and the model was capable of estimating the overall iron-making process. The slight difference in top gas composition is because industrial data contain a slight amount of O_2_ and CH_4_ in top gas, which does not exist in this model.

### 3.3. Effect of H_2_ Injection on Coke Consumption Rate

**Figure 6:**
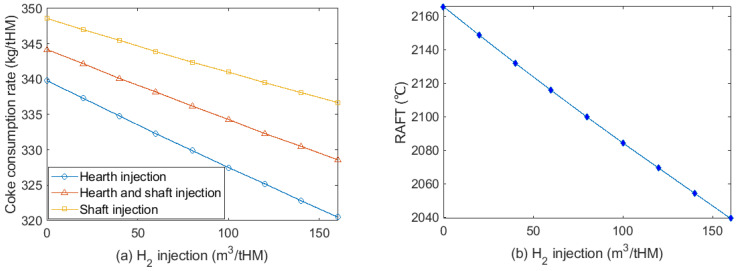
(**a**) Coke consumption rate at different H_2_ injection volumes at different injection positions; (**b**) effect of H_2_ injection to hearth on RAFT at 9% oxygen enrichment rate.

The effect of injecting H_2_-rich gas to hearth and/or shaft on coke rate is shown in Figure 6. The highest H_2_ injection rate is limited to 160 m^3^/tHM to ensure a balanced energy distribution in the BF shaft. Injecting H_2_ to the BF hearth shows less coke consumption than injecting it to the shaft. This is because it provides more sensible heat to the lower part of the furnace to compensate the heat supplied by coke combustion. The relationship between H_2_ injection and coke consumption rate at 9% oxygen enrichment rate is given as in Equation (55). Injecting 7.9~10.9 m^3^/tHM H_2_ can reduce the coke consumption rate by 1 kg/tHM, depending on the injection position.

(55) $$K=351X_{\mathrm{shaft}}+341X_{\mathrm{hearth}}-\left(0.0913X_{\mathrm{shaft}}+0.1275X_{\mathrm{hearth}}\right)\times H_{2\mathrm{inj}}$$

where $X_{\mathrm{shaft}}$ and $X_{\mathrm{hearth}}$ are the proportion of H_2_-rich gas injected to the shaft and hearth, respectively; and $H_{2}$ is the total H_2_ injection volume to the BF, m^3^/tHM.

**Figure 7:**
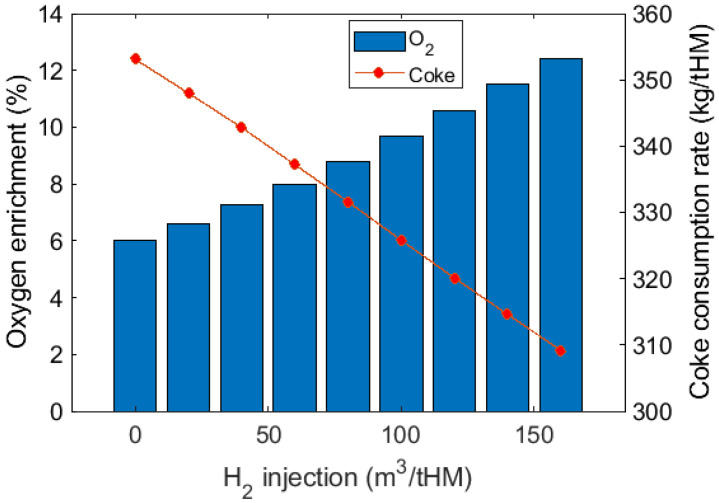
Coke consumption rate at different H_2_ injection volumes at different oxygen enrichment rates for thermal compensation. RAFT was maintained at 2096 °C in this test.

The effect of H_2_ injection on RAFT at a constant oxygen enrichment rate is shown in Figure 6b. Injecting 20 m^3^/tHM H_2_ can reduce RAFT by around 16 °C. Increasing oxygen enrichment can achieve thermal compensation to maintain a stable RAFT and reduce coke consumption, as shown in Figure 7. Compared to the cases without thermal compensation, further reduction in coke consumption is obtained at 309 kg/tHM with 12.4% oxygen enrichment at 160 m^3^/tHM H_2_ injection.

### 3.4. Effect on H_2_ Utilisation Efficiency

**Figure 8:**
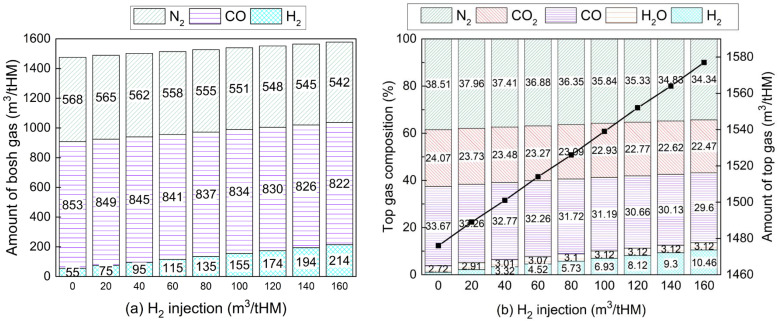
Variations of (**a**) bosh gas and (**b**) top gas composition with hydrogen injection. (H_2_ injection at 0 m^3^/tHM indicates the operating condition of a traditional BF with oxygen enrichment rate at 9%).

**Figure 9:**
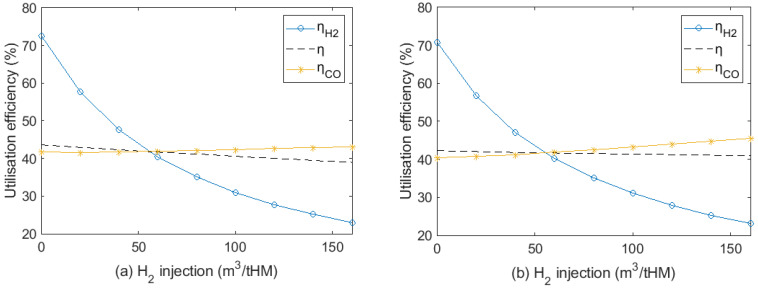
Variations in gas utilisation efficiency with hydrogen injection (**a**) before and (**b**) after thermal compensation.

In the case of injecting H_2_ to hearth, bosh gas composition is shown in Figure 8a. As the H_2_ injection rate increased from 0 to 160 m^3^/tHM at a constant CO injection rate, CO content in bosh gas drops due to less coke consumption. N_2_ content also decreased since less hot blast is required for carbon combustion. There was no H_2_O in the bosh gas because H_2_O that formed from the iron oxide reduction by H_2_ reacted with coke to generate H_2_ again at high temperature. The amount of top gas and its composition is described in Figure 8b. With more H_2_ injection, the moisture content in the top gas increases slightly from 2.72% to 3.12% when the H_2_ injection rate reaches from 0 to 100 m^3^/tHM. This is because H_2_ utilisation efficiency significantly dropped from 72.6% to 22.9%, as shown in Figure 9a. CO content in top gas decreased because of less CO in the bosh gas and increased CO utilisation efficiency. CO_2_ concentration was reduced by less than 2% because there was a significant drop of CO in the bosh gas and increase in CO utilisation efficiency is very limited. The H_2_ injection promotes CO utilisation with the presence of the water–gas shift reaction. However, the overall effect of the water–gas shift reaction was very limited across the whole BF. The comprehensive gas utilisation efficiency gradually decreased from 43.6% to 39.0% when H_2_ injection reached 160 m^3^/tHM.

H_2_ and CO utilisation efficiency after thermal compensation is shown in Figure 9b. Results in this simulation reflect a similar trend as in the literature results [8,49]. Oxygen enrichment increased, and the nitrogen composition in the blast decreased with H_2_ injection. Hence, there was more increase in reducing gas concentration than that in Figure 9a. With less N_2_ dilution and stronger indirect reduction, CO utilisation with thermal compensation was enhanced by 5% with H_2_ injection from 0 to 160 m^3^/tHM.

By H_2_ increasing in the BF, CO utilisation increased, and H_2_ utilisation efficiency decreased. One reason is that water–gas shift reaction Equation (18) would tend to proceed to the right-hand side, and both H_2_ and CO_2_ are generated in the upper furnace below 1000 °C. With the regeneration of H_2_, FeO reduction in Equation (7) can be considered to be proceeding in two successive stages, as shown in Equations (13) and (18):

$$\mathrm{From}\;\mathrm{Equation}\;(13):\;\mathrm{FeO}\;\left(s\right)+H_{2}\;\left(g\right)\to \mathrm{Fe}\;\left(s\right)+H_{2}O\left(g\right)$$

$$\mathrm{From}\;\mathrm{Equation}\;(18):\;H_{2}O\left(g\right)+\;\mathrm{CO}\;\left(g\right)=H_{2}\;\left(g\right)+{\mathrm{CO}}_{2}\;\left(g\right)$$

$$\mathrm{From}\;\mathrm{Equation}\;(7):\;\mathrm{Overall}:\;\mathrm{FeO}\;\left(s\right)+\mathrm{CO}\;\left(g\right)\to \mathrm{Fe}\;\left(s\right)+{\mathrm{CO}}_{2\;}\left(g\right)$$

Due to the smaller size and high diffusivity of H_2_, the reaction in Equation (13) has an advantage over the reaction in Equation (7). Thus, FeO reduction by CO was promoted by increased H_2_ content.

**Figure 10:**
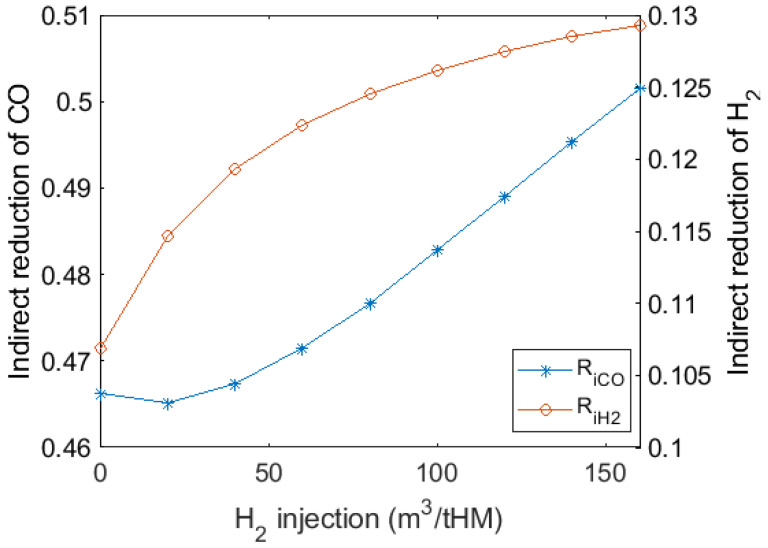
Variations in degree of indirect reduction by CO and H_2_ with H_2_ injection and thermal compensation.

As depicted in Figure 10, with H_2_ injection and thermal compensation, the degree of indirect reduction increased because H_2_ reduction replaced part of the direct reduction and oxygen enrichment enhanced reducing gas atmosphere. Since the direct reduction was a huge endothermic reaction process in the lower furnace, less direct reduction reduces coke consumption. Compared to the higher H_2_ injection volume, injecting 0 to 80 m^3^/tHM H_2_ generates more effect on the degree of indirect reduction, from 0.107 to 0.125. Further, the injection of more than 50 m^3^/tHM H_2_ significantly increased the indirect reduction of CO. Considering gas utilisation efficiency, H_2_ injection should be around 50–80 m^3^/tHM to maintain smooth BF operation and avoid too much excess H_2_ in top gas.

### 3.5. Effects on CO_2_ Emissions and Energy Consumption

**Figure 11:**
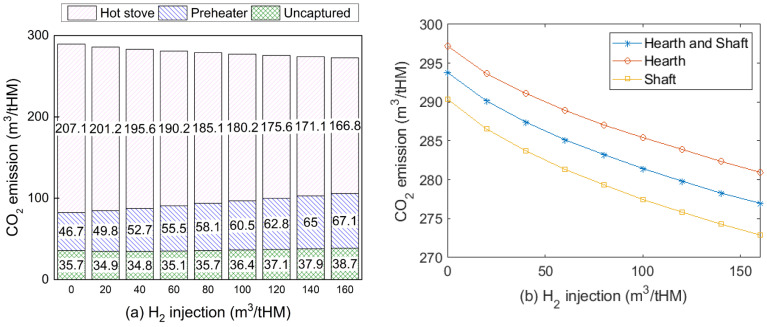
CO_2_ emissions of iron-making process with H_2_ injection (**a**) with different CO_2_ emission sources for shaft injection case; (**b**) at different injection positions (injection temperature for shaft and hearth was 900 and 1250 °C, respectively).

In this work, the CO injected into the BF comes from the CO_2_ captured in top gas, which reduces CO_2_ emission compared to a traditional BF process. The emission from CCU can be negligible because it applies renewable electricity. The total emission of this iron-making process includes uncaptured CO_2_, flue gases from BFG combusted in the preheater and the hot blast stoves, which is shown in Figure 11a. The main CO_2_ emission reduction comes from the hot stoves. Compared with the traditional BF iron-making system that lacks CCU and gas injection, CO_2_ emission dropped from 534 to around 272 m^3^/tHM with 160 m^3^/tHM H_2_ injection in this system. When increasing H_2_ content from 0 to 80 m^3^/tHM, CO_2_ emission only decreased by 20 m^3^/tHM because more BFG was consumed to preheat the injection gas. Additionally, injecting H_2_-rich gas into the shaft showed more CO_2_ emission reduction capability than injecting H_2_ into a hearth or both tuyeres, as shown in Figure 11b.

The energy consumption of the process described in Figure 1 was calculated on the basis of static mass and energy balance, using Equation (56). The standard coal coefficient for each substance in a kilogram of coal equivalent per ton of hot metal (kgce/tHM) is used in this analysis [50].

(56) $$E_{net}=E_{coke}+E_{PC}+E_{BOFG}+E_{blast}+E_{cap}+E_{conv}+E_{water}-E_{export}-E_{O2}$$

where $E_{net}$ is the net energy consumption of the process; $E_{coke}$ is the energy input by coke; $E_{PC}$ is the energy input by pulverised coal; $E_{BOFG}$ is the energy input by BOFG to the hot blast stoves; $E_{blast}$ is the energy carried by the blast to hot blast stoves; $E_{cap}$ is the electricity required for CO_2_ capture unit; $E_{conv}$ is the electricity required for CO_2_ electrolyser, as calculated by Equation (2); $E_{water}$ is the energy carried by the make-up water required at the humidifier for H_2_ generation in the electrolsyer; $E_{export}$ is the energy carried by the BFG that is exported to other processes in the integrated steel mill; and $E_{O2}$ is the energy carried by the oxygen that is generated in the electrolyser and exported to other processes in the integrated steel mill.

**Figure 12:**
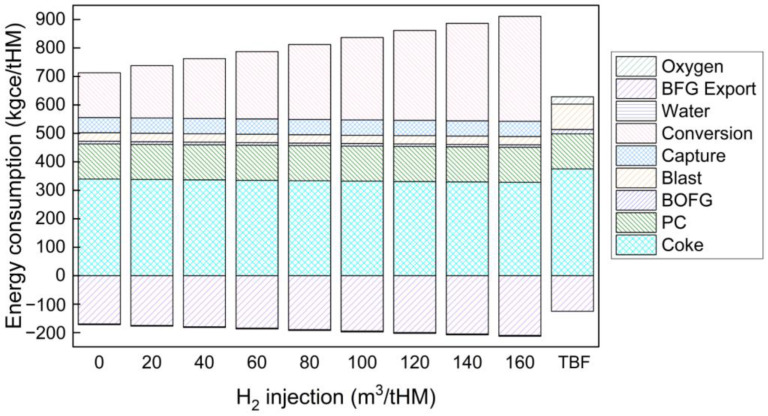
Energy consumption for H_2_-rich gas injection BF and traditional BF process (energy carried by the make-up water needed for the CO_2_ conversion and by BOFG was less than 1% of total energy consumption and thus not indicated in this figure).

Energy consumption results are shown in Figure 12. In a traditional BF, carbon resources from coke and pulverised coal injection provide the primary energy input, which accounts for 80% in total. When hydrogen is injected into the BF from 0 to 160 m^3^/tHM, carbon only accounts for 65% to 50% of the total energy consumption. With H_2_-rich gas injection from 0 to 160 m^3^/tHM, net energy consumption increased from 541 to 698 kgce/tHM, which is higher than that of the traditional BF (504 kgce/tHM) because electricity required for CO_2_ conversion to generate H_2_ kept increasing from 158 to 369 kgce/tHM.

In summary, increasing hydrogen injection volume can reduce coke consumption and CO_2_ emissions. For coke consumption and CO_2_ emission reduction, the hydrogen injection amount should be as much as possible, as long as it satisfies the energy balance in the BF. In this case, the hydrogen injection amount should be 160 m^3^/tHM. However, injecting too much H_2_ significantly reduces its utilisation efficiency and increases the net energy consumption of this process. Further study is recommended to develop a multiobjective optimisation model to balance these effects of hydrogen injection. In general, injecting H_2_ at around 80 m^3^/tHM may consume less energy and suppress CO_2_ emissions under the simulation conditions.

**Figure 13:**
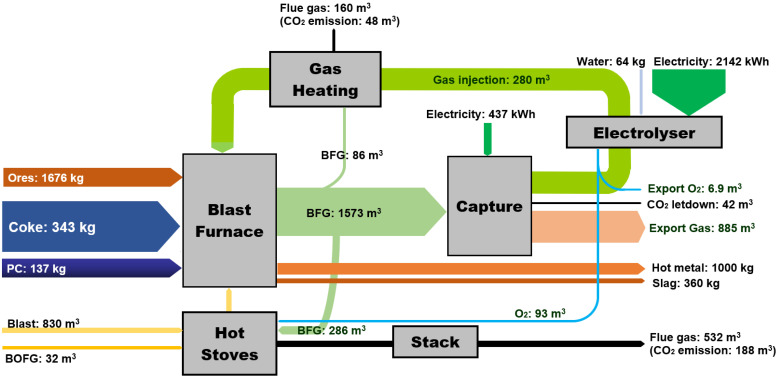
Energy and material consumption of the process with 80 m^3^/tHM hydrogen injection. Relative flow size estimated by energy and demonstrated as bar width.

Energy consumption to produce 1 tonne of hot metal in the case of injecting 80 m^3^/tHM H_2_ with 200 m^3^/tHM CO is indicated in Figure 13. Compared to the traditional BF as indicated by Table 2, coke consumption decreased by 43 kg/tHM. CO_2_ emission dropped from 534 m^3^/tHM for a traditional BF to 278 m^3^/tHM in this case (including gas heating flue gas, capture unit CO_2_ letdown, and stack flue gas). However, electricity consumption in the CO_2_ capture unit and electrolyser is one of the largest energy inputs. The economic impact of this CCU technology as an auxiliary system to the BF is highly recommended for future investigation.

## 4. Conclusions

In this study, a thermodynamic model was used to determine the utilisation efficiency of H_2_-rich gas by considering H_2_ behaviour at different reduction stages. A static mass and energy balance model of the BF was adopted with this thermodynamic model. The effect of injecting H_2_-rich gas on BF performance was determined in terms of its coke consumption, CO_2_ emissions, and energy consumption. Under these simulation conditions, the major findings of this study were:

1. The desired shaft gas injection temperature should not exceed 1000 °C to suppress the endothermic FeO reduction reaction by H_2_-rich gas.
2. Injecting H_2_ to BF hearth has a better effect on coke rate reduction than that of injection to the shaft. The lowest H_2_ consumption to save 1 kg of coke was estimated to be 7.9 m^3^/tHM.
3. H_2_ utilisation efficiency dropped significantly with increasing H_2_ content, and the increase in CO utilisation efficiency was limited. Further research should focus on improving H_2_ utilisation efficiency with a high H_2_ injection rate.
4. Considering H_2_ utilisation efficiency and the degree of indirect reduction by H_2_ and CO, the proper H_2_ injection rate should be from around 50 to 80 m^3^/tHM.
5. Introducing H_2_-rich gas injection can reduce CO_2_ emissions of the iron-making process by up to 262 m^3^/tHM compared with a traditional BF. However, injecting too much H_2_ would hinder CO_2_ emission reduction due to its requirement of preheating outside the BF.
6. The energy consumption of this proposed process was higher than that of the traditional BF. Although coke consumption was reduced by 43 kg/tHM more than that of the traditional BF, net energy consumption increased with the amount of injected hydrogen due to the high electricity consumption in the CO_2_ capture and electrolyser. Developing a CO_2_ conversion unit with higher efficiency but less energy consumption is strongly recommended.

## Figures and Tables

**Table 1:** Operating conditions of the simulation.

Operating Parameters	
PCI rate (kg/tHM)	137
Blast temperature, °C	1052
Humidity of hot blast, g/m^3^	12.93
Top gas temperature, °C	161
Hearth injection temperature, °C	1250
Shaft injection temperature, °C	900

**Table 2:** Quantitative validation of the model.

Parameter	Prediction	Industrial	Top Gas	Prediction	Industrial
Coke rate, kg/tHM	386	386	CO, %	25.1	24.9
Blast, Nm^3^/tHM	1060	1089	CO_2_, %	21.1	20.0
Slag rate, kg/tHM	364	373	H_2_, %	1.2	0.8
Burden input, kg/tHM	1676	1676	N_2_, %	50.0	53
RAFT, °C	2205	2195	R_d_	0.46	-

**Table 3:** Chemical composition of raw material and dust, *w*/*w*%.

Composition	Tfe	FeO	SiO_2_	CaO	MgO	TiO_2_	S	Al_2_O_3_
Sinter 1	55.85	9.59	5.25	10.35	2.19	0.17	0.03	2.5
Sinter 2	55.85	9.2	5.26	10.27	2.2	0.27	-	2.5
Pellet	61.92	1.46	5.31	1.45	-	1.58	-	0.98
Ti ore	40.27	-	9.07	2.05	-	10.78	-	1.69
Dust	39.80	2.58	4.05	2.04	0.83	0	-	1.31

**Table 4:** Chemical composition of coke and pulverised coal, *w*/*w*%.

Composition	Fixed C	H_2_O	FeO	CaO	SiO_2_	Al_2_O_3_	MgO	N	O	H	S
Coke	86.06	4.50	1.6	0.39	4.49	3.69	0.35	0.30	0.21	0.33	1.80
PCI	71.15	0	0.03	1.98	8.4	7.93	0.18	0.34	3.16	2.10	0.30

**Table 5:** Chemical composition of hot metal, *w*/*w*%.

Composition	Fe	C	Si	Mn	P	S	Ti
Hot metal	95.14	4.12	0.34	0.32	0.136	0.023	0.129

## Data Availability

Data sharing not applicable. No new data were created or analysed in this study. Data sharing is not applicable to this article.

## Nomenclature

**Abbreviations**	
BF	Blast furnace
PCI	Pulverised coal injection
CCU	CO_2_ capture and conversion unit
RAFT	Raceway adiabatic flame temperature
tHM	Tonne of hot metal
**Roman and Greek symbols**	
E	Energy, kWh/tHM or kgce/tHM
V	Volume of gas, m^3^/tHM
T	Temperature, °C
$C_{i}$	Specific heat capacity, kJ/m^3^·°C
$K_{i}$	Reaction equilibrium constant of reduction stage i; i = I, II, III
G	Gibbs free energy, kJ/mol
φ	Volume fraction of gas component
$\eta_{i}{}_{\mathrm{CO}}$	Utilisation efficiency of CO in stage i, i = 1, 2, 3
$\eta_{i}{}_{H_{2}}$	Utilisation efficiency of H_2_ in stage i, i = 1, 2, 3
n	Minimal CO required for iron ores reduction, mol
m	Minimal H_2_ required for iron ores reduction, mol
$X_{i}$	Volume reaction of CO or H_2_ in reducing gas entering the BF shaft
η	Overall gas utilisation efficiency for H_2_-rich reducing gas in the BF
%(Reducing gas)	Proportion of H_2_ and CO in the total amount of gas entering the bosh and shaft.
Q	Sensible heat of material, kJ/tHM
H	Enthalpy of material, kJ/tHM
$R_{d}$	Degree of direct reduction
$r_{i}$	Degree of indirect reduction
${\left(\mathrm{Fe}\right)}_{\mathrm{HM}}$	Iron content in hot metal
$\Delta H_{\;}^{0}$	Enthalpy of reaction, kJ/tHM
w	Weight fraction of solid material
$H_{2\mathrm{inj}}$	Total H_2_ injection volume to the BF, m^3^/tHM
